# (Epi)Genetic Mechanisms Underlying the Evolutionary Success of Eusocial Insects

**DOI:** 10.3390/insects12060498

**Published:** 2021-05-27

**Authors:** Kayli R. Sieber, Taylor Dorman, Nicholas Newell, Hua Yan

**Affiliations:** 1 Department of Biology, University of Florida, Gainesville, FL 32611, USA; kayli.sieber@ufl.edu (K.R.S.); taylor.dorman@ufl.edu (T.D.); nicholas.newell@ufl.edu (N.N.); 2 Center for Smell and Taste, University of Florida, Gainesville, FL 32611, USA

**Keywords:** epigenetics, eusocial insects, evolution, behavioral plasticity

## Abstract

**Simple Summary:**

Social insects, namely ants, bees, and termites, are among the most numerous and successful animals on Earth. This is due to a variety of features: highly cooperative behavior performed by colony members and their specialization on a variety of tasks. Diverse physiological and behavioral specializations are regulated not only by the genetic system, but also by the epigenetic system which alters gene expressions without modifying the genetic code. This review will summarize recent advancements in such studies in eusocial insects.

**Abstract:**

Eusocial insects, such as bees, ants, and wasps of the Hymenoptera and termites of the Blattodea, are able to generate remarkable diversity in morphology and behavior despite being genetically uniform within a colony. Most eusocial insect species display caste structures in which reproductive ability is possessed by a single or a few queens while all other colony members act as workers. However, in some species, caste structure is somewhat plastic, and individuals may switch from one caste or behavioral phenotype to another in response to certain environmental cues. As different castes normally share a common genetic background, it is believed that much of this observed within-colony diversity results from transcriptional differences between individuals. This suggests that epigenetic mechanisms, featured by modified gene expression without changing genes themselves, may play an important role in eusocial insects. Indeed, epigenetic mechanisms such as DNA methylation, histone modifications and non-coding RNAs, have been shown to influence eusocial insects in multiple aspects, along with typical genetic regulation. This review summarizes the most recent findings regarding such mechanisms and their diverse roles in eusocial insects.

## 1. Introduction

Sociality is a key feature of many of Earth’s most successful animal species. Living in packs, herds, or groups has a variety of advantages that culminate to improve the inclusive fitness (Table 1) of an individual. While members of many animal groups (namely arthropods and vertebrates) possess social species, only a few select groups can be considered “eusocial.” These groups possess several features that distinguish them from other groups of social creatures, which include overlapping generations within a society, cooperative brood care, and division of labor into reproductive and non-reproductive groups [1]. Eusocial insects, including those of the orders Hymenoptera (ants, bees, and wasps) and Blattodea (termites), are among the most well-studied eusocial animals. 

The division of labor exhibited by eusocial insects has resulted in the specialization of society members, with many species exhibiting different “castes” that perform specific tasks or behaviors. In many cases, castes are rigid and determined during development [38,39]. In other cases, individuals may undergo behavioral changes, performing different tasks over time. This is particularly well-documented in the honeybee, *Apis mellifera* [40,41]. In other cases, individuals may switch their caste due to external cues, and even achieve reproductive status when they were formerly non-reproductive as observed in the ant species *Harpegnathos saltator*, the Indian jumping ant [42,43]. In most eusocial insects, caste determination and behavior are not dictated by heritable genetic information [44], further evidencing the flexible nature of the eusocial insect phenotype and calling for a better understanding of how such differences arise in this widely successful animal group. Genetic diversity within a colony is typically very low, and in some cases colony members may even be genetically identical. The array of phenotypes within a single colony along with colony-wide genetic similarity suggests that diversity is likely a result of changes in an individual’s gene expression, not differences in the genetic code itself, and differences in gene expression play an important role in eusocial insect colony structure and function. 

The study of epigenetics (Table 1) focuses on molecular changes (particularly in regard to gene expression) unrelated to changes in the genetic sequence [3]. Specifically, this field aims to understand the molecular mechanisms and developmental processes occurring to establish diverse phenotypes without a change in genotype [4,5]. The epigenetic changes are mitotically heritable over the course of an organism’s life, but the degree to which these changes are inherited by offspring differs depending on the type of organism. In some groups (such as plants) epigenetic features are highly heritable across generations (transgenerational epigenetic inheritance) [45,46], whereas they are less heritable in mammalian groups [46,47]. 

Epigenetic system responds to changes in the environment. Environmental signals induce activation of sensory neurons and internal hormonal responses, which in turn modulate signal transduction pathways and activity of transcription factors (TFs). TFs recruit epigenetic factors and turn on the expression of target genes (reviewed in [48,49,50,51]). Even when the environmental signal is no longer present, the changes in hormonal composition or target gene expression may be maintained, suggesting that epigenetic processes could be at play in the maintenance of molecular changes induced by the environment [48,49,50]. 

Epigenetic mechanisms involve modifying histones (Table 1), as in the addition of acetyl group, methyl group, phosphate group, or ubiquitin protein [6,7,8], and adding side groups to DNA, as in methylation (Table 1), the process of adding a methyl group to a cytosine nucleotide [14,15,16]. These modifications control gene expression by condensing and relaxing chromatin (Table 1), a complex of DNA and histone proteins, thereby altering the accessibility of genes for transcription. Non-coding RNAs (ncRNAs) also play prominent roles in epigenetic regulatory control and alteration of gene expression: microRNAs (miRNAs) (Table 1), for example, are small ncRNAs (~22 nucleotides in size) that are involved in translational suppression through binding to messenger RNA (mRNA) [23]; long non-coding RNAs (lncRNAs) (Table 1) are greater than 200 nucleotides in length, and their most notable regulatory role has been determined in HOX gene expression, dosage compensation, genomic imprinting, etc. [24,25,26]. Varying epigenetic processes may also interact with one another. The lncRNA HOTAIR, for example, is known to recruit polycomb repressive complex 2 (PRC2), which catalyzes histone H3K27 methylation. The ncRNA *Xist* is involved in X-chromosome inactivation, recruiting chromatin modifiers to induce heterochromatin formation, and silencing the whole chromosome, providing another example of the interaction of multiple mechanisms [25]. It is important to keep in mind that a single mechanism is often not acting alone. However, mechanistic interaction in eusocial insects has not been heavily examined.

Epigenetic mechanisms have been shown to regulate caste determination, aging, reproduction, behavior, and other categories in eusocial insects (as reviewed in [49,50,52,53,54,55]), but there is still much to be gained from further study that can aid in building a clearer picture of eusocial insect life history and evolution. This review primarily aims to summarize recent advancements in our understanding of these mechanisms, their implications, and how they apply to the evolutionary history and success of eusocial insects.

## 2. Caste Determination, Plasticity, and Caste-Specific Behavior

Eusocial insects exhibit the ability to generate more than one phenotype from a single genome. Castes within a single colony may differ morphologically and behaviorally to such a degree that they visually appear to be different species. If genetically similar or identical individuals can develop such differing morphological and behavioral forms, there is basis for the assumption that different gene expression patterns must be established during the developmental period. What role might the insect epigenetic system have in caste determination and plasticity?

One of the defining factors of eusociality is reproductive division of labor [1]. Most members of a colony are non-reproductive and few produce fertile eggs. Typically, reproductive capability is determined in the juvenile (larval) state. Considering how fundamental this form of caste differentiation is to eusocial insect societies, understanding how it occurs from an epigenetic perspective is important for bettering our understanding of eusocial insect societies. 

**Figure 1:**
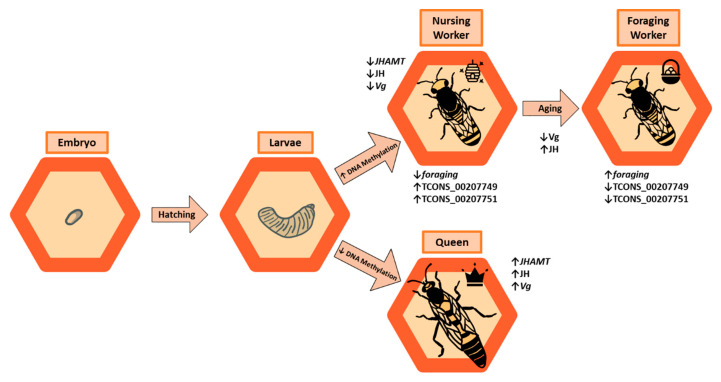
Epigenetic modifications occur at different points in the *A. mellifera* life cycle. Embryos hatch into larvae, which may develop into workers or queens. Workers overall possess a higher level of methylation than queens, opposing the case in bumblebees and ants. Honeybee workers also possess decreased expression of JH synthesis genes. As workers age, they switch from a nursing behavioral phenotype (signified by the hive icon) to a foraging behavioral phenotype (signified by the basket icon). This switch is associated with decreased Vg and increased JH production, as well as expression differences in the *foraging* gene and associated lncRNAs.

DNA methylation, catalyzed by members of the DNA methyltransferase (DNMT) family (Table 1), is an important factor in caste determination. Unlike mammals and other vertebrates, most eusocial insect species exhibit lower levels of DNA methylation [56,57] and those areas which are methylated tend to be gene body regions [57,58]. Still, it seems to be an important feature of caste determination. Silencing *dnmt3* (Table 1), a gene involved in de novo DNA methylation, results in *A. mellifera* larvae developing into reproductive queens instead of non-reproductive workers [59]. When fed diets high in methionine, a methyl donor, *A. mellifera* larvae tend towards development into workers [60], and the effects of methionine appear to be neutralized when coupled with a DNA methylation inhibitor [60]. Therefore, higher levels of methylation induce development of the worker phenotype, while having comparatively less methylation induces a queen phenotype (Figure 1). 

Many Hymenopteran species are understudied, and whether methylation is involved in their caste determination process is unknown. Recent efforts expanded this area of research into DNA methylation-related genes in the bumblebee, *Bombus terrestris*. Caste-specific patterns were identified for DNMTs, methyl-CpG-binding domain proteins (MBDs, which recognize methylated sites for recruitment of repressive chromatin modifiers) and ten-eleven translocation proteins (TETs, which are DNA demethylases). Reproductive tissues in queens exhibit high expression of most of these genes, except for *TET* [61], implying a need for higher methylation levels in bumblebee queens. One hundred and eleven differentially methylated genes have been identified between castes of *B. terrestris*, including genes involved in processes related to reproduction [62]. In the narrow-headed ant *Formica exsecta*, DNA methylation occurs to a higher degree in queens as opposed to workers [63]. These findings align with previous work in the red harvester ant *Pogonomyrmex barbatus* [64]. This trend of increased methylation in ant and bumblebee queens opposes the notion in honeybees that increased methylation results in a worker phenotype, suggesting that the relationship between DNA methylation and reproductive development may differ across the Hymenoptera. However, the supposed role of methylation in the queen phenotype has not been verified. Differing levels of DNA methylation may also result from different genetic backgrounds, seasonal factors, or age, which should be taken into consideration when concluding whether methylation is truly important for development of queen and worker castes. Indeed, the role of DNA methylation in eusocial insect caste determination is a debated subject (a thorough review in [65]). Furthermore, it is possible that certain DNMTs have functions aside from methylating DNA (such as the role of DNMT1 in the beetle *Tribolium castaneum* [66]). 

It is important to note that DNA is not the only nucleic acid which can be modified. RNA can also undergo modifications which impact gene expression post-transcriptionally [67]. In eukaryotes, *N*^6^-methyladenosine (m^6^A) (Table 1) is a modification applied to mRNA to serve a variety of functions, including regulation of RNA processing and translation [21,22]. The RNA m^6^A methylome of *A. mellifera* was recently reported, and workers were shown to have a higher number of m^6^A-modified transcripts than queens. Notably, *juvenile hormone acid O-methyltransferase* (*JHAMT*) transcripts had higher methylation in worker larvae [68] (Figure 1). JHAMT is a component of the juvenile hormone (JH) biosynthesis pathway, regulating the activation of this well-established honeybee caste determinant [69]. Worker larvae also had elevated m^6^A levels on transcripts for *vitellogenin* (*Vg*), a JH antagonist [68] (Figure 1). Queen-like features (namely increased JH levels) resulted from chemical suppression of m^6^A marks by 3-deazaadenosine (DAA) during the larval stage [68]. As of now, these marks have only been studied in a single species, *A. mellifera*. Given that m^6^A seems to impact caste determination and development in this species, it will be important to expand this work to other species to determine whether RNA modification is a universal caste determinant. 

DNA/RNA methylation is not the only modification involved in caste determination. Acetylation of histone H3 on lysine 27 (H3K27ac) (Table 1), a modification associated with transcriptional activation, exhibits caste bias in honeybee larvae. In queen larvae, H3K27ac is localized within exons and around transcriptional start sites, while it is located in introns of worker larvae [70]. 

**Figure 2:**
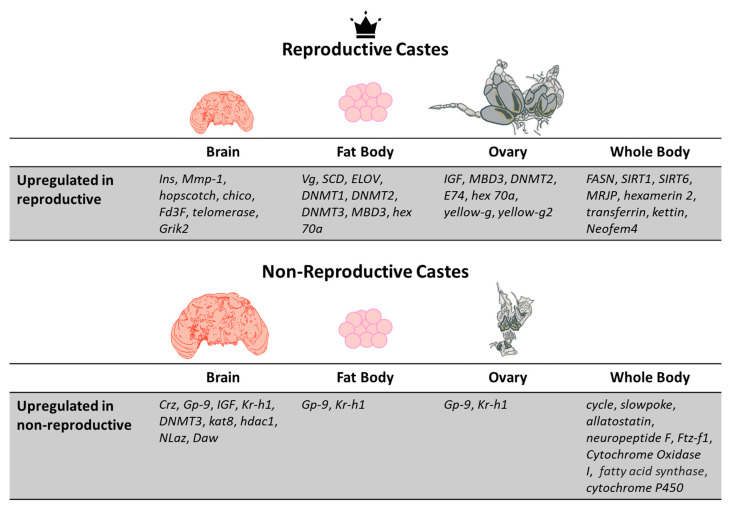
A summary of the upregulated genes and structural changes occurring in three different tissue types (the brain, fat body, and ovary) in reproductive and non-reproductive castes. Additionally, genes found to be upregulated in transcriptome analyses of whole insect bodies are also included. Non-reproductive females possess larger brains and inactivated ovaries, while reproductive females generally experience a reduced brain size, but much larger activated ovaries. Representative genes from all major eusocial insect lineages are listed here, including genes from ants [31,32,33,34,37,73,74], bees [75,76,77,78,79,80], social wasps [81,82], and termites [72].

Similar to the Hymenoptera, termite colonies also share similar genetic backgrounds and are able to form varied castes, suggesting that gene expression differences are responsible for phenotypic differences in this group as well. This has been studied in the termite species *Reticulitermes labralis*, a species in which workers become reproductive upon isolation. Differentially expressed genes (DEGs, exhibiting upregulated or downregulated expression between groups) have been identified between isolated and non-isolated workers [71], suggesting that dynamic changes in gene expression are involved in caste transition. Older work in the dampwood termite *Zootermopsis nevadensis* also exemplifies gene expression differences between reproductive and non-reproductives, showing that *Vg* and *Neofem4* are upregulated in reproductives (Figure 2) [72]. Adult reproductive plasticity of this type also notably occurs in the Hymenoptera and will be discussed in detail in Section 4. 

The primary discussion of this section has been on the differentiation between royals and workers. Many eusocial insect species have caste structures of much greater complexity, possessing several non-reproductive castes. Recently, it was shown that miRNAs influence termite soldier caste development. Eight differentially expressed miRNAs have been identified among the non-reproductive castes of *R. speratus*, three being up-regulated in workers and five being up-regulated in soldiers [83]. Four differentially expressed miRNAs (mir-87a, 2765, 133, and 125) are shared between honeybees [84] and termites [83], suggesting that differential expression of miRNAs between castes may be an important factor in the evolution of sociality and caste structure across different lineages. However, miRNAs known to be associated with queen development in *A. mellifera* are not differentially expressed in *B. terrestris*. In contrast, two miRNAs, Bte-miR-6001-5p and Bte-miR-6001-3p, are highly expressed in queen-destined *B. terrestris* larvae [85]. These two species share a primitively eusocial common ancestor [86], suggesting that the differences in their caste-specific miRNA expression must have arisen after their evolutionary divergence. 

In species with castes that are both morphologically and behaviorally distinct, these traits are generally fixed in adulthood. However, they can be artificially manipulated, and one caste may be forced to “switch” its behavioral phenotype, despite being morphologically suited for alternative tasks. In *Camponotus floridanus*, the Florida carpenter ant, workers can be divided into the major caste (which performs defensive behaviors) and minor caste (which performs nursing and foraging behaviors). A histone post-translational modification, H3K27ac, is involved in regulating expression of genes involved in this caste identity, aiding in the generation of distinct phenotypes from a common genotype [87]. Majors can be reprogrammed to foraging via administration of histone deacetylase inhibitors (HDACi’s) (Table 1), shifting behavioral phenotypes towards the minor form as acetylation levels are increased [88]. Corepressor for element-1-silencing transcription factor (CoREST) also experiences upregulation when HDACi is administered, and subsequently promotes JH synthesis by repressing JH-degrading enzyme production [89]. These findings imply the involvement of CoREST and acetylation in the development of these two distinct castes, though there has not yet been a study to validate this possibility. 

Histone acetylation also appears to be involved in circadian rhythm (also known as the sleep-wake cycle) in eusocial insects. Circadian rhythm may be determined by task specialization, age, and social context [90,91], and differs between castes. Foraging insects, for example, must leave the nest at different times of the day to collect food. Nurses and queens, on the other hand, may experience different circadian activity due to their remaining inside the nest. Histone acetyltransferase (HAT) (Table 1) inhibition eliminates circadian rhythmicity almost entirely in workers of the ant species *Temnothorax longispinosus* [92], a reminiscence of studies in mammals that transcription of circadian rhythm-related genes are dependent on histone acetylation [93,94]. Currently it is unknown whether acetylation plays a unanimous role in the circadian rhythm of eusocial insects. 

In summary, caste determination is a fundamental process essential to the formation of the typical eusocial insect colony structure. While many factors play a role in caste determination, the role of epigenetic mechanisms in such morphological and behavioral diversity deserves further examination. 

## 3. Reproduction and Juvenile Development

Within a colony of eusocial insects, the number of reproductive individuals is typically limited to a single or a small number of queens. In the case of termites, there is additional presence of a king. Spermatogenesis and oogenesis, the processes yielding the two types of gametes needed for sexual reproduction, may experience epigenetic influence that could impact the resulting offspring in a manner independent of offspring genotype. 

DNA methylation likely influences gametogenesis in eusocial insects. A study of caste-specific gene expression patterns in the bumblebee *B. terrestris* found TET2 expression in drone testes to be very high [61], suggesting a need for demethylation in the process of sperm production. Conversely, DNMT3, the de novo DNA methyltransferase, is highly expressed in testes of the termite *R. speratus* [95] and in ovaries and embryos of the fire ant *Solenopsis invicta* [96]. High levels of DNMT1 (Table 1), the maintenance DNA methyltransferase, are present in testes of *S. invicta* [96]. This raises the possibility that different forms of methylation are required in testes and ovaries of different species. In *S. invicta* for example, methylation marks might be maintained in sperm during cell division. In ovaries and embryos, on the other hand, epigenetic reprogramming (Table 1) might occur via establishment of new methylation marks. Further study in *B. terrestris* has illustrated a relationship between methylation and egg production of queens. Treatment with a methylation inhibitor results in higher egg production by queens, and differential methylation of loci involved in oogenesis [97]. However, as most insects are featured with gene body methylation which is not associated with transcriptional repression, it is not clear how induced methylation differences modify gene expression in bumblebees. Furthermore, the aforementioned study did not examine the expression levels of any identified differentially methylated genes. This is an area of research worthy of more focus. 

Most eusocial insects are rigid in their eusociality, except for a few facultatively eusocial species such as the *Megalopta genalis* sweat bee. Females of this species nest either socially or solitarily, so they are good candidates for comparative studies and may be a reasonable model for eusocial evolution. Female gene expression differs between eusocial and solitary nesting phenotypes and shows correlation to developmental gene expression changes [98]. Understanding how gene expression differs between social and solitary groups could add a significant component to our knowledge of the underlying changes that have led to advanced sociality and nesting behavior.

After fertilization and egg-laying, further epigenetic modifications related to hormones and molting periods take place. RNA interference (RNAi) of *histone methyltransferase 4–20* (*Hmt4-20*) in *Z. nevadensis* resulted in extended developmental stages. This is likely due to delayed JH action, as expression of JH synthetic genes and JH signaling genes were decreased in *Hmt4-20* RNAi [99], and the hormone itself is critical for termite development and caste determination [38,100]. Evidently, epigenetic mechanisms have significant influence over insect development. The gene targeted in the aforementioned study has only been examined in the context of termites, and so similar research should investigate other members of the Hymenoptera to identify epigenetic mechanisms potentially controlling JH synthesis and molting periods. 

## 4. Age-Dependent Behavior, Aging, and Longevity

Eusocial insects display variable lifespans among castes, with queens living notably longer than workers. Differential lifespan in queens vs. workers may arise without genetic change, and instead under epigenetic control, modifying phenotype without influencing genotype [101]. In addition, multiple environmental factors may modulate epigenetic system and gene expression, thereby altering lifespan of individuals within the same caste. 

Environmental factors, such as seasonality, impact longevity in honeybees. In warmer seasons, honeybees live only a few weeks. During the winter season, honeybees from the same hive may live for months. Differences in gene expression are likely involved in this stark difference, suggesting epigenetic involvement. The hypopharyngeal glands of honeybees are responsible for secreting royal jelly proteins, important for queen development [102]. In captive honeybee colonies, workers are put in an induced low-activity state to help their overwintering. Consequentially, the hypopharyngeal glands are suppressed during the winter, while they are activated in warmer months by colony activity [103]. DNA methylation may facilitate the restoration of atrophied hypopharyngeal glands, specifically regulating epidermal growth factor receptor (EGFR) and forkhead box protein O (FOXO) genes [104]. 

Parasites are also capable of impacting longevity in ants. *Temnothorax nylanderi* ants are an intermediate host to the tapeworm species *Anomotaenia brevis*. Infected workers display increased fecundity and longevity comparable to that of a queen, even though they are not of a royal caste [105]. Queens and infected workers share high expression of one known anti-aging gene, *carboxypeptidase B* [106], which has a suggested role in delayed senescence [107]. Enhanced lifespan of infected workers may also be due to overexpression of immunity-related genes, although there is little overlap between immunity gene expression in queens vs. infected workers [106]. Future research may benefit from examining the relationship between increased fecundity and longevity in infected workers to better understand the physiological changes that occur when a worker is parasitized. 

**Figure 3:**
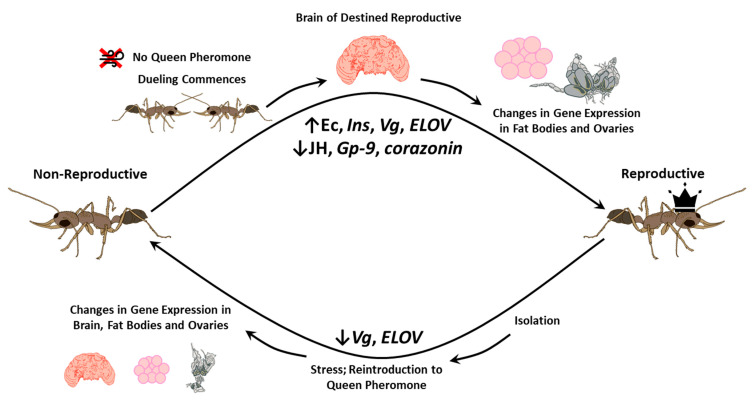
*Harpegnathos saltator* workers undergo changes in gene expression and tissue structure to become reproductive gamergates. In the absence of queen pheromones, workers will commence dueling, a behavior in which antennal strikes are rapidly exchanged between workers. Victors will become destined reproductive. Changes in gene expression in the brain will trigger gene expression changes in fat bodies and ovaries, eventually resulting in reproductive status. The gamergate state is not permanent and can be reversed following isolation and subsequent introduction to the pheromone of another reproductive. Changes in gene expression and tissue structure undergo reversion, and the gamergate behaves like a regular worker once again. The brain figure is adapted from Smith et al., 2016 [109], and the ovary images are adapted from Gospocic et al., 2017 [32].

Non-reproductive *H. saltator* ant workers may undergo transition into fertile pseudoqueens (“gamergates”) (Table 1) without experiencing genomic change. The transition is accompanied by a change in nervous cell composition, e.g., with gamergates experiencing a ~40% increase in neuroprotective ensheathing glial cells in the brain [73]. This change in cellular composition may contribute to an increased longevity in gamergates, fivefold in comparison to workers, by allowing the brain to actively respond to damage as the individual ages [73]. In addition, a damage responsive gene *Mmp-1* is upregulated in gamergates, providing a molecular mechanism responsible for their longer lifespan [73]. Gene expression changes in the ovaries and fat bodies occur, preluded by initial gene expression changes in the brain of the transitioning individual. These changes included increased ecdysone, *Ins*, *ELOV*, and *Vg*, and decreased JH, *corazonin*, and *Gp-9* [33] (Figure 3). Interestingly, gamergates can be reverted to regain worker phenotypes [108]. The gamergate transition involves a reduction in brain size and activation of the ovaries, but when reverted, the original brain size is restored, and the ovaries are inactivated once again. Gamergates exhibit fertility-signaling cuticular hydrocarbons (CHCs) and a reduction in venom gland size, features that also revert when gamergates switch back to a worker phenotype. Changes in gene expression (notably decreased *Vg* and *ELOV* expression) also occur during reversion [108] (Figure 3). 

The process of aging in eusocial insects varies among different species and castes, and in some cases behavioral changes are associated with age. *A. mellifera* honeybees undergo a behavioral switch as they age, transitioning from performing tasks within the nest, such as nursing, to performing tasks that involve leaving the nest, such as foraging. This is associated with hormonal fluctuations, namely a decrease in Vg and an increase in JH levels (Figure 1), which promotes the foraging behavior [110]. There may be a relationship between methylation and this hormonal aspect of aging, as inhibiting DNMTs in mature honeybees results in increased Vg levels and longevity [111]. This suggests that Vg acts independently of JH, with increased levels generating a longer lifespan regardless of JH levels. While there appears to be a connection between methylation, Vg levels, and longevity, it is not understood how methylation affects Vg. 

The age-dependent behavioral switch from nurse to forager is thought to be associated with lncRNAs TCONS_00207749 and TCONS_00207751, which target the *foraging* gene for inhibition. *foraging* has higher expression in foragers than in nurses and helps to regulate the behavioral transition from one state to the other [112]. Consistent with this, TCONS_00207749 and TCONS_00207751 have low expression in foragers [113], allowing *foraging* to attain the higher expression levels seen in foraging behaviors (Figure 1). This suggests a correlation between these lncRNAs and age-dependent behavioral plasticity, and it is assumed that lncRNAs might be responsible for the inhibition of *foraging* during the young nursing stage and their lower expression may be responsible for *foraging* expression during the older foraging stage. However, functional analysis is not performed yet to address the role of lncRNAs in age-dependent behavioral transition.

## 5. Social Communication

Eusocial insects coexist in groups that often populate into thousands and millions of individuals. Social stimuli, such as pheromones and other volatile molecules, dominance behavior, and presence of brood can all impact the behavior and functional status of the colony. Although behavioral consequences of social interactions have been well-studied in vertebrates [114,115], there is much yet unknown in eusocial insects. In better understanding the molecular nature of eusocial insect interactions and social context, we may gain insight into their evolution and their highly cooperative nature.

Eusocial insect colony structure is somewhat plastic in that the relative proportion of brood to workers varies over time. Colony dynamics have profound effects on different aspects of *A. mellifera* physiology, including impacting longevity and worker behavior [116,117,118,119,120,121]. Findings have illustrated a relationship between DNMTs and social context in *A. mellifera* workers [122]. Brood presence affects *dnmt3* and *dnmt1* transcript levels, suggesting that some DNA methylation may be occurring in a manner dependent upon colony composition [122]. 

Much of the communication that occurs within a colony is a result of pheromones and other volatile chemicals (chemical communication is reviewed in [51], and the evolution of the insect olfactory system is reviewed in [123]), and it is worth noting that *dnmt3* expression is associated with queen-related pheromonal cues in honeybees as well as in ants [124]. Queen mandibular pheromone (QMP) manipulates a variety of physiological traits of honeybee workers, including reproductive inhibition [125,126], learning [127], and various aspects of behavior [128,129]. QMP action may depend on epigenetic mechanisms in the brains of worker honeybees, as expression of *dnmt3* and histone modifier genes (such as *lysine acetyltransferase 8*, aka *kat8*, associated with acetylation, and *histone deacetylase 1*, aka *hdac1*, associated with deacetylation) increase in worker brains when exposed to QMP (Figure 2) [130]. Further studies should investigate how queen-related and brood-related pheromones impact worker DNA methylation levels, and thus may impact different aspects of worker physiology and colony dynamics. 

## 6. Neural Tissue and Functionality

Eusocial insect behavior and caste systems are influenced by a variety of factors, many of which have already been discussed above in this review. The interconnectedness of these factors leads to epigenetic regulation of genes within the nervous tissues of eusocial insects. 

Epigenetic mechanisms acting at different developmental stages are responsible for differential brain development in *A. mellifera* workers and queens [131]. *tum*, *mnb*, *Tor*, and *insulin receptor 1* (*InR-1*) genes are expressed at greater rates in workers than queens during development. In contrast, *insulin-like growth factor* (IGF, see Table 1) is expressed more in queens during the same phase [131]. Notably, *tum* and *mnb* have known neurogenic function [132,133], while RNAi knockdown of *Tor* has been shown to induce a worker phenotype by reducing JH levels in queen-destined larvae [134]. Differential expression of certain genes during development leads to morphological distinctions between worker and queen brain structures [131]. 

Altered gene expression in the brain is also associated with the dramatic caste switching observed in *H. saltator* gamergates. Expression of *corazonin* in the brain is notably downregulated and *insulin* (*Ins*, see Table 1) level in the brain is increased, along with a global decline in JH levels. Decreased JH levels and lowered *corazonin* expression contribute to increased *Vg* expression in reproductive tissues [33] (Figure 2 and Figure 3). The change in *Ins* levels is also supported by work in the clonal raider ant *Ooceraea biroi*, in which worker ovaries can be strongly activated using *Ins* supplementation [34]. Notably these changes in gene expression likely reflect the need to alter the phenotype of the ant from worker to reproductive pseudoqueen, and alterations to the molecular workings of the brain may be key components of this transition. Some of these genes, such as *corazonin* and *Ins*, are likely conserved DEGs related to reproductive capabilities in different eusocial insect groups.

The activation and inhibition of DNA methylation in honeybees can affect learning and odor memory [135], and have been linked to long-term memory formation and re-learning [136,137,138]. Examination of mushroom bodies (Table 1) in *A. mellifera* has shown a positive relationship between methylation levels and olfactory learning [139]. 

DNA methylation in honeybee neural tissue is likely a powerful determinant of behavior. Behavioral diversity in *A. mellifera* is of great interest in the entomological community due to the drastic differences in aggression between honeybee subspecies. Africanized honeybees (*A. m. scutellata*) are far more aggressive than the European (or western) honeybee. Previous work illustrated differential expression of aggression-related genes in the brains of Africanized honeybees and other subspecies [140], as well as methylation differences between subspecies [141]. In European honeybees, the brain undergoes molecular changes and altered gene expression when aggressive behavior is necessary [142,143,144,145,146]. Aggression-specific methylation profiles in European honeybees were recently reported, also providing the first evidence of an epigenetic component of aggression in bees [147]. Interestingly, some of the differential methylation resulting from aggressive behavior in European honeybees overlaps with differential methylation between the Africanized and European subspecies [141,147]. This suggests possible conserved epigenetic regulation of aggressive behaviors. 

miRNAs are thought to play a critical role in eusocial evolution by participating in regulation of socially important traits. Evidence suggests that social and solitary bee species express different neural miRNAs [148]. One hundred and fourteen and ninety-seven miRNAs have also been identified in brain tissues of the Formosan subterranean termite *Coptotermes formosanus* and *R. speratus* termites, respectively [149]. The miRNAs miR-11-3p and miR-13b-3p, found in both species, are among the most upregulated [149]. These miRNAs target genes important for neural function, including *Comm2*, *fra*, *FucTA*, *Ara*, *Cas*, and other genes [149,150,151,152,153]. It is still not understood how miRNAs expressed by the brain affect transcription of genes linked to eusociality, providing basis for future study. 

lncRNAs have also been shown to function in eusocial insect nervous systems. In *H. saltator* and *C. floridanus*, 438 and 359 nervous tissue lncRNAs have been identified, respectively [154]. A few of the lncRNAs identified in brain tissue include XLOC_044583, XLOC_109542, XLOC_001194. The first is associated with various brain regions, and the other two with the non-visual brain and the optic lobe, respectively [154]. These lncRNAs could potentially play a role in development and function of the nervous system in eusocial insects.

Future research should compare the epigenetic modifications occurring in neural tissue in eusocial and solitary species, as well as queens and workers of eusocial species. For example, recent comparative analysis of the transcriptomes of queen, male, gyne, and worker brains in the pharaoh ant *Monomorium pharaonis* identified where they differ (Figure 2) [37]. Studies such as these are important for building the foundation for future studies of epigenetic mechanisms. To gain a better understanding of the epigenetic pathways regulating neural plasticity, epigenome and transcriptome profiles should be established using brain tissue from workers, queens, and males from both eusocial and solitary insect species, followed by functional analysis, e.g., in model eusocial insects (as below). Only then can we begin to understand insect nervous system development and regulation.

## 7. Transgenerational Epigenetic Inheritance

Epigenetic modifications and molecular mechanisms play well-established roles in affecting gene function and regulation in eusocial insects, as well as in other invertebrates and vertebrate animals. However, whether epigenetic modifications (e.g., DNA methylation) are heritable across generations in insects is still debated. In mammals, methylation marks are erased and re-established during early embryo development [28,155]. This pattern hinders epigenetic inheritance in mammalian reproduction, though it does occur to a small extent. Whether a similar pattern occurs consistently across insect groups remains to be determined. Previous studies have suggested parental effects in social insects [57,156,157,158]. However, there has been little evidence to prove that transgenerational epigenetic inheritance is specifically responsible for these observed parental effects. 

It is well-established that the level of DNA methylation observed in eusocial insects is relatively lower than what is observed in vertebrates [56,57], and primarily located at gene body regions, which are not associated with gene silencing [57,58]. Studies on epigenetic remodeling and methylation reprogramming in invertebrates (namely in *A. mellifera*) have shown methylation marks to remain relatively stable during embryogenesis, suggesting that eusocial insects may differ from vertebrates in their ability to maintain DNA methylation marks across generations [159,160]. 

Patriline differences have been shown to affect worker characteristics, including reproductive traits [161], suggesting that there are heritable paternal effects being passed down from drone to worker. While these differences could have an exclusively genetic basis, honeybee drones also possess individual-specific patterns of DNA methylation in their semen, raising the possibility that patriline-specific methylation patterns could be inherited by a drone’s daughters [162]. In the case of honeybees, male drones are haploid, and thus all the sperm of a drone is genetically identical. In assuming that epigenetic marks in honeybees are not reprogrammed during embryogenesis, the epigenetic marks present in a drone’s semen should not undergo extensive change from the point of fertilization on through the development of the daughter worker. Consequentially, a drone’s daughters should share 100% of their paternal methylome with their sisters. Indeed, there is evidence that is consistent with this notion, as workers share a high proportion of methylated sites with their fathers in a patriline-specific manner, differing in methylation patterns from their half-sisters [163]. While this provides evidence for heritable epigenetic effects in honeybees, these effects are only intergenerational (across two generations). For these effects to be considered transgenerational, further study needs to prove their heritability across multiple generations. 

Little focus has been placed on matriline-specific methylation and worker inheritance of queen epigenetic marks. It is possible that workers inherit some maternal methylation marks. However, if no reprogramming occurs, queens would be expected to give rise only to queens in offspring. Yet, honeybee queens give rise to workers who possess higher levels of methylation. Queen and worker caste development seems to rely to some extent on different levels of methylation and de novo methylation by DNMT3, as discussed previously in the body of this review [58,59]. These ideas suggest that some degree of epigenetic reprogramming likely occurs in eusocial insects, at least in a matriline-specific manner. 

While honeybees may inherit epigenetic marks from their parents, whether there a true lack of reprogramming is debatable. Evidence from work in *S. invicta* suggests that some level of erasure and reprogramming occurs in Hymenopteran development. *dnmt3* is highly expressed in ovaries and in embryos of this ant [96], as well as in testes of termite *R. speratus* [95]. Why would this methyltransferase be present in gametogenesis unless de novo methylation were occurring? Perhaps methylation marks are erased and rewritten during gametogenesis, but with near-complete fidelity. This may give the illusion that these insects lack developmental reprogramming. Alternatively, perhaps reprogramming does not occur in early embryo development, but rather later in the larval stage to result in queen and worker phenotypes. Finally, it is possible that DNMT genes play roles beyond methylation. Perhaps observed *dnmt* expression is not a sign of methylation, but rather these genes serve some other role in reproduction that has not been identified in eusocial insects. This is evidenced by studies in non-eusocial species, such as the red flour beetle *Tribolium castaneum*, a species with little to no observable DNA methylation that still expresses *dnmt1* through its entire life cycle. Knockdown of the gene in beetle mothers is associated with high offspring mortality [66]. Given the lack of methylation in this species in conjunction with the apparent necessity of *dnmt1*, it is suggested that DNMT genes may serve multiple roles in insects. *dnmt1* has also been shown in other insects to play a potential role in female fecundity and in embryo survival, including in one species of wasp [164,165,166]. Further study from this perspective may yield interesting and novel results.

## 8. Eusocial Insects as Models

Through the pioneering efforts of early researchers in the field, genetic and epigenetic studies of eusocial insects have become more numerous and easier to perform as our understanding of the field has improved. Establishment of genetic tools in eusocial insects continues to expand into new species. The CRISPR/Cas9 system has notably been established in three ant species, *H. saltator* [167,168], *O. biroi* [169], and most recently in the fire ant *S. invicta* [170]. CRISPR/Cas9 [171,172,173,174,175,176] and transgenesis [177,178] have also been established in the honeybee *A. mellifera*. Such tool development and its application in functional studies are foundational for future molecular work, as continued progress of epigenetic studies in eusocial insects depends on constant improvement of our manipulability of enzymes that catalyze epigenetic modifications. Precise epigenome editing has been widely used in mammals (reviewed in [179,180]). With continued development of genetic tools, similar approaches will be applied to eusocial insects in the future. 

Eusocial insects exhibit arguably the highest degree of social organization of any animal group. Furthermore, they exhibit incredible potential for serving as models for epigenetic modifications and mechanisms. Due to their plasticity (i.e., the ability to develop a variety of castes within a single colony) and the increasing number of species which can be kept in labs, they present opportunities for unique research focused on the evolution of social behavior and the phenotypic diversity differentiating these groups from other insects, studies which cannot be done in other insect groups. While these insects present complex phenotypes, they are relatively simple systems in terms of body structure and neuroanatomy, evidencing the benefits of using such organisms for studies that may be more difficult to perform in complex mammalian model systems. Continuing to sequence genomes and transcriptomes of these insects will provide novel targets for functional analysis using genetic and epigenetic tools in diverse eusocial insects, allowing for better insights into the mechanisms underlying eusociality. 

## 9. Conclusions

The last several years have seen important advancements in our understanding of epigenetic mechanisms in eusocial insects. Indeed, scientists have made breakthroughs in a variety of entomological and genetic subfields, expanding our knowledge of the molecular underpinnings of eusocial insect development, neuroscience, and behavior. Now more than ever, research is illustrating the importance of epigenetics in deciphering the unique features exhibited by eusocial insects, such as their diverse caste structures as well as their plasticity in reproductive capabilities and longevity. It is this natural plasticity, along with the increasing ease of rearing social insects in laboratory settings, that makes this group suitable for epigenetic study. 

Despite continual advancements in our understanding of eusocial insects, there are still many questions remaining to be answered. Caste determination appears to be regulated at least in part by epigenetic factors, but how conserved this regulation is across species remains to be seen. Of particular interest is the ability of some species to transition from non-reproductive to reproductive caste, extending a lifespan several times longer than that of a normal worker. The molecular mechanisms underlying this transition are of growing interest and considerable importance to aging research. Other notable unanswered questions involve whether epigenetic modifications are heritable across generations, and whether Hymenopterans and termites undergo any epigenetic reprogramming during development. Additionally, it is worthwhile to continue studying epigenetic modifications from a behavioral perspective, as such study could be beneficial to ecology and pest management. Given the continual expansion of this field into more species, the rich new findings that are being made, and the increasing ease of performing such studies, epigenetic research in eusocial insects will continue developing into a fruitful field. 

## Figures and Tables

**Table 1:** Terminology associated with epigenetic study in eusocial insects.

Term	Definition	Reference(s)
Inclusive fitness	A measurement of fitness in which the success of an animal is derived from the summation of an animal’s own reproductive fitness and of cooperative or altruistic behaviors exhibited by genetically similar individuals.	[2]
Eusociality	The highest degree of sociality exhibited by animals. Distinguished by overlapping generations in a colony, cooperative brood care, and division of labor.	[1]
Epigenetics	The study of changes in traits unrelated to changes in the genetic code. Such traits are mitotically heritable (through cell division).	[3,4,5]
Histone modification	The addition of an acetyl group, methyl group, phosphate group, or ubiquitin protein to histone proteins.	[6,7,8]
H3K27ac	Acetylation of histone H3 on lysine 27, a histone modification associated with transcriptional activation.	[9]
HAT	Histone acetyltransferase that transfers acetyl groups to lysine amino acids.	[7,10]
HDAC	Histone deacetylase for removal of acetyl groups from histones.	[11]
HDACi	Histone deacetylase inhibitors.	[12,13]
DNA methylation	Addition of a methyl group to a cytosine nucleotide.	[14,15,16]
DNMT family	The DNA methyltransferase family of proteins that are responsible for catalyzing DNA methylation.	[17,18]
DNMT1	The maintenance DNA methyltransferase.	[19]
DNMT3	The de novo DNA methyltransferase.	[20]
N^6^-methyladenosine	A form of RNA methylation, which has functions in RNA regulation.	[21,22]
miRNAs	microRNAs are non-coding RNAs of around 22 nucleotides in length. They suppress translation by binding to mRNA.	[23]
lncRNAs	Long non-coding RNAs are non-coding RNAs longer than 200 nucleotides. They have variable functions.	[24,25,26]
Chromatin	A complex of DNA and histone proteins which may be modified to be condensed or relaxed, thereby suppressing or promoting gene expression.	[27]
Epigenetic reprogramming	Erasure and rewriting of histone marks and DNA methylation.	[28]
Gamergate	A pseudoqueen: lack of queen pheromone in the colony induces workers to achieve reproductive status.	[29]
Mushroom body	The region of the insect brain responsible for olfactory and visual learning and memory functions.	[30]
*IGF*	Homolog of *insulin-like growth factor* in mammals, also called *Ilp-1* in *Apis mellifera* and *Ilp-2* in *Harpegnathos saltator.*	[31,32,33]
*Ins*	Homolog of mammalian *insulin*, also called *Ilp-1* in *Harpegnathos saltator*, *Ilp-2* in *Apis mellifera* and *Ooceraea biroi*, and *LIRP* in *Monomorium pharaonis*.	[31,32,33,34,35,36,37]
